# The epidemiological survey of *Coxiella burnetii* in small ruminants and their ticks in western Iran

**DOI:** 10.1186/s12917-022-03396-0

**Published:** 2022-07-28

**Authors:** Maryam Rahravani, Meysam Moravedji, Ehsan Mostafavi, Mehrdad Mohammadi, Hamid Seyfi, Neda Baseri, Mohammad Mahdi Mozoun, Mina Latifian, Saber Esmaeili

**Affiliations:** 1 Department of Clinical Sciences, Sanandaj Branch, Islamic Azad University, Sanandaj, Iran; 2 National Reference Laboratory for Plague, Tularemia and Q Fever, Research Centre for Emerging and Reemerging Infectious Diseases, Pasteur Institute of Iran, Akanlu, Kabudar Ahang, Hamadan, Iran; 3 Department of Epidemiology and Biostatics, Research Centre for Emerging and Reemerging Infectious Diseases, Pasteur Institute of Iran, Tehran, Iran

**Keywords:** *Coxiella burnetii*, Q fever, Tick, Sheep, Goat, Iran

## Abstract

**Background:**

Q fever is one of the most important zoonotic diseases caused by *Coxiella burnetii*. Although Q fever is an endemic disease in Iran, epidemiological data on *C. burnetii* infection are not yet complete in reservoirs and vectors in some parts of Iran. This survey investigated *C. burnetii* infection in small ruminants (sheep and goat blood samples) and their ticks in western Iran (Kurdistan province) in 2020. The presence of *C. burnetii* DNA was identified in these samples by targeting the *IS1111* gene using the quantitative PCR (qPCR) method.

**Results:**

Out of 250 blood samples (232 sheep and 18 goats), *C. burnetii* was detected in two samples (0.8%) belonging to the sheep (0.9%). In addition, 34 of 244 collected ticks (13.9%) from infested animals (244) were positive for *C. burnetii* infection. The highest prevalence of infection was found in *Dermacentor marginatus* (18.3%) and *Haemaphysalis concinna* (12.5%).

**Conclusions:**

The present study showed that ticks could have a possible role in the epidemiology of Q fever in Iran.

**Supplementary Information:**

The online version contains supplementary material available at 10.1186/s12917-022-03396-0.

## Background

Q fever is one of the most important zoonotic diseases caused by an obligate intracellular bacterium called *Coxiella burnetii*. *C. burnetii* is a worldwide pathogenic agent which is endemic in all countries except New Zealand [1]. The host range of *C. burnetii* is diverse, including mammals (wild and domestic), birds, reptiles, and arthropods. Livestock such as cattle, sheep, and goats are the main reservoirs of this bacterium. Although *C. burnetii* infection is usually asymptomatic or subclinical in animals, abortion, stillbirth, premature delivery, weak offspring, infertility, metritis, and mastitis have been reported in some cases [2, 3]. Shedding of this bacterium into the environment could occur through milk, urine, feces, mucosal secretions, and birth fluids by infected animals [1].

*C. burnetii* are mainly transmitted to humans by inhalation of infected aerosols. In humans, the clinical manifestations of Q fever are diverse, from asymptomatic infection to acute and chronic Q fever [4]. Asymptomatic infection can occur in more than 60% of patients. Acute Q fever is usually a self-limited illness characterized by non-specific symptoms such as fever, headache, myalgia, chills, fatigue, pneumonia, and hepatitis [5]. Chronic Q fever has different manifestations, including endocarditis, vasculitis, lymphadenitis, osteomyelitis, and spontaneous abortions [1–3]. In case of improper treatment, Q fever endocarditis can lead to severe or a life-threatening illness [1, 3].

Among arthropods, ticks are the main host and vector of *C. burnetii* [6]. More than 40 species of ticks can be naturally infected by *C. burnetii* [6]. *Ixodes*, *Rhipicephalus*, *Dermacentor*, and *Haemaphysalis* genera are the most prevalent hosts and vectors of this bacterium [7]. Ticks can play a role in *C. burnetii* transmission to animals, but human infections by tick biting are rarely reported [1, 4].

In the recent decade, several seroepidemiological studies among human and domestic animals in different parts of Iran indicated that Q fever is an endemic disease in Iran. According to a systematic review and meta-analysis study in Iran, the estimated seroprevalence of the Q fever among humans, cows, sheep, and goats were 19.8%, 13.3%, 24.7%, and 32%, respectively [8]. Also, the overall prevalence of *C. burnetii* in milk samples of cows, sheep, goats, and camels was estimated at 15.1%, 3.8%, 7.85, and 1.4%, respectively [9]. On the other hand, acute Q fever cases and Q fever endocarditis have been reported in Iran in recent years [10–15].

The epidemiology of Q fever is poorly understood in some parts of Iran, especially in western provinces. Based on the only study in Kurdistan province, the seroprevalence of Q fever was very high (27.8%) among the different human populations [16].

No study has been conducted on livestock in this province, and no data is available in this regard. Without information on the status of *C. burnetii* infection in animals (especially domestic animals) and ticks, it is impossible to understand the Q fever situation in the area. Therefore, this survey aimed at investigating the prevalence of *C. burnetii* infection in small ruminants and their ticks in Kurdistan province.

## Results

In this study, 250 blood samples (232 sheep and 18 goats) were collected from 33 small ruminant flocks (Table 1). The prevalent gender was female in both sheep (93.1%) and goats (94.4%). The mean age (± SD) of sheep and goats were 3.31 (± 1.4) and 4.78 (± 1.8), respectively. The age of small ruminants was categorized into three sub-groups: Age 0–1 year: 33; 2–3 years: 90; > 3 years: 127.

**Table 1:** Detection of *C. burnetii* in blood samples of small ruminants in Kurdistan Province

	**Sheep**		**Goat**	
	**N (%)**	**No positive for *C. burnetii* (%)**	**N (%)**	**No positive for *C. burnetii* (%)**
**Sanandaj**	4 (1.7)	0 (0)	0 (0.0)	ND
**Baneh**	15 (6.5)	0 (0)	0 (0.0)	ND
**Marivan**	46 (19.8)	0 (0)	0 (0.0)	ND
**Divandarreh**	167 (72)	2 (1.2)	18 (100)	0 (0)
**Total**	232	2 (0.9)	18	0 (0)

*ND* Not defined

Two hundred and forty-four ticks were collected from small ruminants (Table 2). Among 244 ticks, 229 (93.8%) and 15 (6.2%) ticks were collected from sheep and goats, respectively. One hundred and twenty-five (51.2%) ticks were male, and 119 (48.8%) were female. *Dermacentor marginatus* was the most prevalent (67.2%) tick among collected samples. Other collected ticks included *Rhipicephalus sanguineus* sensu lato (10.7%), *Rhipicephalus turanicus* (12.3%), and *Haemaphysalis concinna* (9.8%)*.*

**Table 2:** Detection of *C. burnetii* among collected ticks from small ruminants in Kurdistan province

	**No of tested ticks (no. positive, %)**	**No of positive ticks based on host (%)**		**No of positive ticks based on county**^a^ **(%)**			
		**Sheep**	**Goat**	**S**	**M**	**B**	**D**
***Rhipicephalus sanguineus* sensu lato**	26 (0, 0)	0 (0)	ND	0 (0)	0 (0)	0 (0)	0 (0)
***Rhipicephalus turanicus***	30 (1, 3.3)	1 (3.33)	ND	1 (100)	0 (0)	0 (0)	0 (0)
***Haemaphysalis concinna***	24 (3, 12.5)	2 (12.5)	1 (12.5)	ND	ND	1 (33.3)	2(8.33)
***Dermacentor marginatus***	164 (30, 18.3)	26 (16.6)	4 (57.1)	ND	0 (0)	1 (33.3)	29 (18.5)
**Total**	244 (34, 13.9)	29 (12.7)	5 (33.3)	1 (50)	0 (0)	2(18.2)	31 (16.7)

^a^*S* Sanandaj, *B* Baneh, *M* Marivan, *D* Divandarreh. *ND* Not defined

All individual qPCR results of *C. burnetii* in tick and small ruminant blood samples are reported in Supplementary Information file 1. *C. burnetii* was detected in2 out of 232 (0.9%) sheep blood samples. Both positive samples belonged to the same flock in Divandarreh County. No positive cases were found among goat blood samples.

In total, 34 of 244 collected ticks (13.9%) were positive for *C. burnetii* using qPCR (Table 2). The prevalence of *C. burnetii* among *D. marginatus*, *R. turanicus*, and *H. concinna* were 18.3%, 3.3%, and 12.5%, respectively. No positive samples were detected in *R. sanguineus* sensu lato. The prevalence of *C. burnetii* in collecting ticks based on hosts was 12.7% in sheep and 33.3% in goats. In addition, 16.7% of collected ticks from Divandarreh County were positive for *C. burnetii.* No positive tick was found in collected ticks from Marivan County.

## Discussion

This study was carried out for molecular detection of *C. burnetii* in small ruminants and their ticks in western Iran. The presence of *C. burnetii* has been shown in the sheep blood samples (0.8%) and collected ticks (13.9%) from goats and sheep in Kurdistan province. Q fever, caused by *C. burnetii*, is a zoonotic disease of great public health importance worldwide that its prevalence is highly variable from one country to another, due to epidemiological differences and whether or not the disease is reportable [17, 18]. The most notable epidemiological profiles of Q fever, including the hyperendemicity situation, major outbreak, and epidemic were reported in Africa, the Netherlands, and French Guiana, respectively [17]. In 2005, an outbreak of Q fever occurred in 58% of Marines deployed to Iraq, as a western neighbor of Iran. Dust and exposure to animals and ticks were reported as possible risk factors for infection [19]. Human seroprevalence of Q fever was found to be 12.3–32%in Turkey [20, 21]. Moreover, sheep, goats, and herds seroprevalence was reported at 10.5–14.19%, 10.24%, and 44.7%, respectively, in Turkey, a northwestern neighbor of Iran [22, 23]. There is very strong evidence of *C. burnetii* infections in humans and animals in all parts of Iran. However, Q fever is not considered important by the health care system and Veterinary Organization in Iran. There is no control program and surveillance system for Q fever at veterinary and medical levels in Iran. This limitation is a challenge and gap in public health based on available current data. Most studies in Iran, including in Kurdistan province have been conducted on livestock or dairy products [24, 25]. High seroprevalence of *C. burnetii* was reported in sheep (35.9%), goats (56.7%), and cows (32.2%) in the Kurdistan province. Moreover, In this study, 6.5% of collected milk samples from Kurdistan province were positive for *C. burnetii* using the PCR method [26]. Living close to livestock farms and consumption of unpasteurized milk and dairy products have been reported as the risk factors for Q fever [27]. It seems that Q fever is an endemic disease in some parts of Iran, including Kurdistan province. In a study (2011–2012), the seroprevalence of Q fever was 27.8% among different human populations in this province [16]. So that cases of Q fever endocarditis have been recently reported in this province [17]. Acute Q fever was also diagnosed in many cases among suspected patients in the north of Iran indicating a high prevalence of this disease [27]. In Lorestan province, west of Iran, the seroprevalence of Q fever among butchers and slaughterhouse workers was 23.5% [28]. In a meta-analysis in Iran (2017), the most prevalent *C. burnetii* IgG phases I and II antibodies was reported in the provinces of Kerman (24%), in the south-central part of Iran, and South Khorasan (54%), in eastern Iran [8]. Although studies in different regions of Iran warned about the possible outbreak of Q fever in Iran, there has been no comprehensive research on arthropod vectors of *C. burnetii* in Iran, including Kurdistan province. Therefore, understanding the roots of infection in the *C. burnetii* lifecycle in Iran is an important objective.

Domestic ruminants, the main reservoir of *C. burnetii*, are the most important source of Q fever infection in humans. In our study, *C. burnetii* bacteremia was detected in 0.9% of sheep blood samples using the qPCR method. In general, the periods of *C. burnetii* bacteremia in the reservoirs are very short and after a few days, this bacterium localized in the mammary glands, uterus, and placenta. After localization, the *C. burnetii* is shed into the environment via milk, feces, and vaginal discharge for a long time [2]. Therefore, the low prevalence of *C. burnetii* in sheep blood in the current study indicates short-term bacteremia. No molecular positive sample was found in goat blood samples. In addition to short *C. burnetii* bacteriemia, the failure to find positive cases in goat blood samples in this study could be due to the very small number of samples tested.

In this study, 12.7% and 33.3% of collected ticks from sheep and goats were infected by *C. burnetii*, respectively. Overall, 13.9% of collected ticks from these small ruminants were positive for *C. burnetii*.

The highest prevalence of infection was found in *D. marginatus* (18.3%) and *H. concinna* (12.5%). These results suggested that *D. marginatus* may likely have a role to be one of the main ticks in the *C. burnetii* lifecycle in Kurdistan. Interestingly, in both sheep infected with *C. burnetii*, their ticks (*D. marginatus*) were also *C. burnetii* positive. However, a comparison of *C. burnetii* strains in sheep with those from ticks gotten from the sheep was not done.

In the present study, the prevalence of *C. burnetii* in ticks was higher than in other studies in Iran. Furthermore, infected tick species (*D. marginatus*, *R. turanicus*, and *H. concinna*) by *C. burnetii* in this study were not reported in the previous study in Iran. In a study in Sistan and Balouchestan province (southeast Iran), 7.4% of collected ticks from sheep and goats were positive for *C. burnetii,* and infected ticks belonged to the *Hyalomma anatolicum*, and *R. sanguineus* sensu lato [29]. Furthermore, in another study in Sistan and Balouchestan province, 4.8% of collected ticks from cattle were infected by the causative agent of Q fever, and positive ticks belonged to *Hyalomma excavatum*, *H. anatolicum*, and *R. sanguineus* sensu lato [30]. In Kerman province, 11.4% of collected ticks from sheep and goats were positive for *C. burnetii,* and positive ticks belonged to the *H. anatolicum* and *R. sanguineus* sensu lato [31]. In Ardabil province (northwest Iran), 12.5% of collected ticks from goats were infected by *C. burnetii*, and positive ticks belonged to the *H. excavatum*, *H. anatolicum*, and *R. sanguineus* sensu lato [32]. Using the finding of the present study and similar studies, it is assumed that ticks may likely a role in the *C. burnetii* lifecycle in Kurdistan. Therefore, it suggests that studies on *C. burnetii* should be performed among a wide range of ticks and other arthropods to establish the possible role of ticks in the epidemiologic cycling or maintenance of *C. burnetii* in Iran, especially in this endemic area of Iran.

## Conclusions

In conclusion, *C. burnetii* has been found in the sheep blood (0.8%) and body ticks of goats and sheep (13.9%) in western Iran. This evidence may indicate the probable circulation of *C. burnetii* in ticks in Kurdistan province. Therefore, it is supposed that ticks may play a role in the natural lifecycle and epidemiology of this bacterium in western Iran. Although there is considerable evidence of *C. burnetii* infections in humans and animals in Iran, there is a complete lack of control programs and surveillance systems for Q fever at veterinary and medical levels in Iran. The present study suggests that the health care system and veterinary organizations in Iran should be aware of the presence of Q fever disease in the country and the role of ticks in the epidemiologic cycling or maintenance of *C. burnetii* must be assessed in Iran.

## Methods

### Study area

**Fig. 1:**
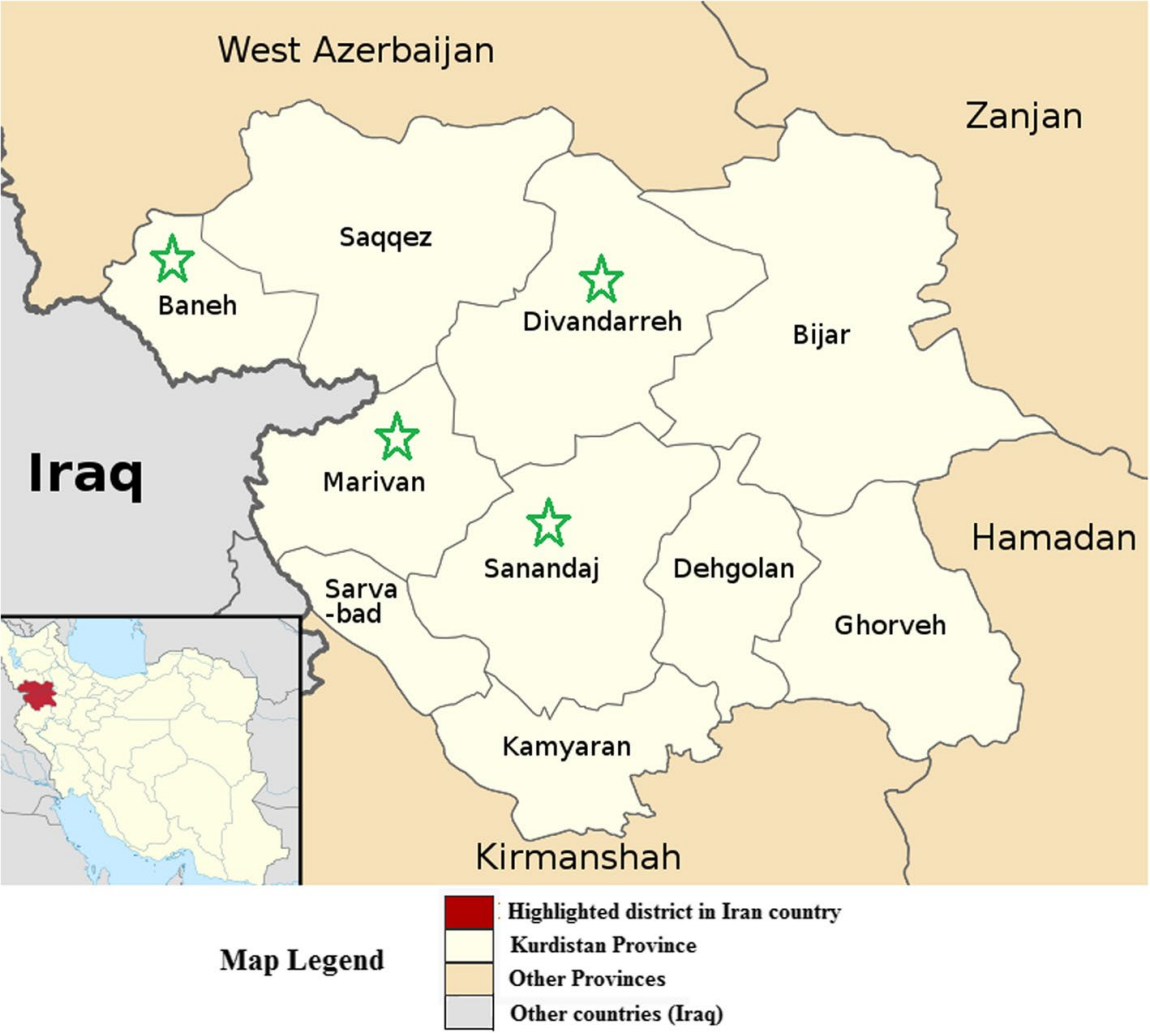
The present study was conducted in four counties of Kurdistan province, including Marivan, Baneh, Sanandaj, and Divandarreh. The location of these counties on the district map of Kurdistan province is indicated with a green star

This study was conducted in Kurdistan province from August to September 2020. Kurdistan province is located in the west of Iran with 28,817 Km^2^ in the area and is bordered by Iraq (from the west). This province is a mountainous region with a cold and harsh climate in winter and autumn and warm and dry weather in the summer season. The human population of this province is estimated at 1.6 million people. In addition, the total number of livestock in Kurdistan province is about 1.417 million animals, including 115,000 cows, 1,077,000 sheep, and 224,000 goats. This study was conducted in four counties (Marivan, Baneh, Sanandaj, and Divandarreh) of Kurdistan province (Fig. 1). These counties were randomly selected.

### Blood sampling and tick collection

We calculated the sample size of small ruminants based on 20% prevalence, 95% confidence interval (CI), and 5% error. According to these values, the minimum required sample was obtained to be 246 samples. Therefore, we included 250 samples in this study. Two hundred and fifty sheep (232) and goats (18) were selected from 33 small ruminant flocks. From each herd, about 1–33 sheep and goats were randomly selected based on the consent of herd owners and available animals. The herds were also randomly selected from each selected county without any considerable conditions. Two hundred and fifty animals were carefully examined for tick infestation, and if they were infested, they were included in the study. One tick per infected animal with ticks was removed and collected in a separate tube. Six (2.4%) animals did not have any ticks on their skin surface. Overall, 244 ticks were collected from 244 animals.

All demographic information is included in supplementary information file 1.

Five milliliters of whole blood were taken by venoject tubes containing anticoagulant (EDTA). After blood sampling of selected animals, one tick per animal was randomly removed from selected animals with fine-pointed, stainless-steel tweezers. Each tick was placed in a separate encoded 2 ml tube, and 70% ethanol was added to each tube. Collected tick samples were transferred to the Parasitology Laboratory of the Veterinary Faculty of the Islamic Azad University of Sanandaj and were morphologically identified using taxonomic keys [33, 34]. For DNA extraction and qPCR experiments, all blood samples and identified ticks were sent to the National Reference Laboratory for Plague, Tularemia, and Q fever of the Research Centre for Emerging and Reemerging Infectious Diseases in Pasteur Institute of Iran under cold conditions.

### DNA extraction from blood samples

The genomic DNA was extracted from the blood samples using a FavorPrep™ Blood/Cultured Cell Genomic DNA Extraction Mini Kit (Favorgen Biotech Corp., Taiwan), following the manufacturer’s instructions. Extracted DNA was stored at -20 °C until molecular analysis. Briefly, 20 µl Proteinase K and 200 µl FABG Buffer were added to a 200 µl whole blood sample and mixed by pulse-vortexing. After incubation at 60 ºC for 15 min, 200 µl ethanol (96–100%) was added to the sample. The mixture was transferred to the FABG Mini Column and centrifuged at 6,000 × g for 1 min. Washing steps were performed with 450 µl of W1 Buffer and 750 µl Wash Buffer. To elute total DNA, 100 µl elution of solution was dispensed into the membrane center of the FABG Mini Column and centrifuge at full speed for 1 min.

### DNA extraction from ticks

Before the beginning of the DNA extraction from salivary glands and midguts of ticks, the following orders were performed respectively: washing their body surface body with 70% ethanol for 5 min, 5% sodium hypochlorite for 5 min, and sterile distilled water for 15 min. After passing the mentioned steps, the body of tick was opened under a loop by a sterile scalpel to remove the salivary glands and midguts. After removal, the organs were placed individually into a ceramic mortar approximately liquid Nitrogen to tissue dryness. Dried tissues were transferred separately to sterile 1.5 ml Microtubes for DNA extractions. The genomic DNA extraction from collecting tick tissues was performed using a commercial kit (G-spin™ tissue DNA extraction kit, iNtRON Biotechnology, South Korea) protocol. Briefly, 200 μl Buffer CL, 20 μl Proteinase K, and 5 μl RNase A Solution were added to the sample tube. After vortexing, the lysate was incubated at 56℃ for 30 min. When lysis was completed, 200 μl of Buffer BL add was added, mixed, and incubated at 70℃ for 5 min. Then the mixture was centrifuged at 13,000 rpm for 5 min. Then 400 μl of the supernatant was mixed with 200 μl of absolute ethanol. The mixture was transferred to the Spin Column and centrifuge at 13,000 rpm for 1 min. After the washing steps, DNA was eluted with 50 μl of elution buffer. Extracted DNA was kept at -20 °C.

### qPCR for detection of *C. burnetii*

*IS1111* element of *C. burnetii* was targeted by qPCR using specific primers ([forward: AAAACGGATAAAAAGAGTCTGTGGTT] and [reverse: CCACACAAGCGCGATTCAT]) and probe (6-FAM-AAAGCACTCATTGAGCGCCGCG-TAMRA) sequences [35]. Sequences of used primer/probe, qPCR mixture, and cycling conditions for qPCR performed during this study are presented in Supplementary Information file 1. Briefly, the final volume of each qPCR reaction was 20 μl, contained 10 μl of 2X Real Q Plus Master Mix for Probe (Ampliqon, Denmark), 1 μl of a mixture of probe (with the final concentration of 200 nM), and forward and reverse primers (with the final concentration of 900 nM), 4 μl of extracted DNA, and 5 μl of double-distilled water (DDW). Amplifications were performed on the Corbett 6000 Rotor-Gene system (Corbett, Victoria, Australia). The qPCR program was 10 min at 95 °C, followed by 45 cycles of 15 s at 94 °C and 60 s at 60 °C. DDW and purified DNA of the Nine Mile strain (RSA 493) were used as negative and positive controls, respectively. qPCR results were analyzed using a Rotor-Gene® Q 2.3.5 software (QIAGEN), and samples were considered positive when showing cycle threshold (Ct) values of 40 or lower.

### Statistical analysis

Data were analyzed with SPSS statistical software, version 22 (SPSS Inc, Chicago, IL, USA). Logistic regression, Pearson's chi-squared, and x^2^ tests were used to compare the variables; *p*-values ≤ 0.05 were considered statistically significant.

## Supplementary Information

**Additional file 1.** The cycling conditions for qPCR performed during study "The epidemiological survey of Coxiella burnetii in small ruminants and their ticks in western Iran.

## Data Availability

The datasets used and/or analyzed during the current study are available from the corresponding author on reasonable request.

## Abbreviations

*C burnetii*: *Coxiella burnetii*
qPCR: Quantitative polymerase chain reaction
DDW: Double-distilled water
Ct: Cycle threshold
*D. marginatus*: *Dermacentor marginatus*
*R. turanicus*: *Rhipicephalus turanicus*
*H. concinna*: *Haemaphysalis concinna*
*R. sanguineus*: *Rhipicephalus sanguineus*
*H. anatolicum*: *Hyalomma anatolicum*
*R. sanguineus sensu lato*: *Rhipicephalus sanguineus sensu lato*
*H. excavatum*: *Hyalomma excavatum*
